# Physiological essay on *Gulliver’s Travels*: a correction after three centuries

**DOI:** 10.1007/s12576-018-00655-4

**Published:** 2019-01-04

**Authors:** Toshio Kuroki

**Affiliations:** 1 Research Center of Science Systems, Japan Society for the Promotion of Science (JSPS), 5-3-1, Kojimachi, Chiyoda-cu, Tokyo, 102-0083 Japan; 2 University of Tokyo, Tokyo, Japan; 3 Gifu University, Gifu, Japan

**Keywords:** *Gulliver’s Travels*, Scaling, Power law, Food requirement, Kleiber’s law, Quetelet’s law

## Abstract

*Gulliver’s Travels* by Jonathan Swift, published in 1726, was analyzed from the viewpoint of scaling in comparative physiology. According to the original text, the foods of 1724 Lilliputians, tiny human creatures, are needed for Gulliver, but the author found that those of 42 Lilliputians and of 1/42 Brobdingnagians (gigantic human creatures) are enough to support the energy of Gulliver. The author further estimated their heartbeats, respiration rates, life spans and blood pressure. These calculations were made by the use of three equations, i.e., body mass index (BMI = *W*/*H*^2^) and quarter-power laws (*E*∝*W*^3/4^ and *T*∝*W*^1/4^), where *W*, *H*, *E*, and *T* denote body weight, height, energy and time, respectively. Their blood pressures were estimated with reference to that of the giraffe and barosaurus, a long-neck dinosaur. Based on the above findings, the food requirement of Gulliver in the original text should be corrected after almost three centuries.

## Introduction

**Fig. 1:**
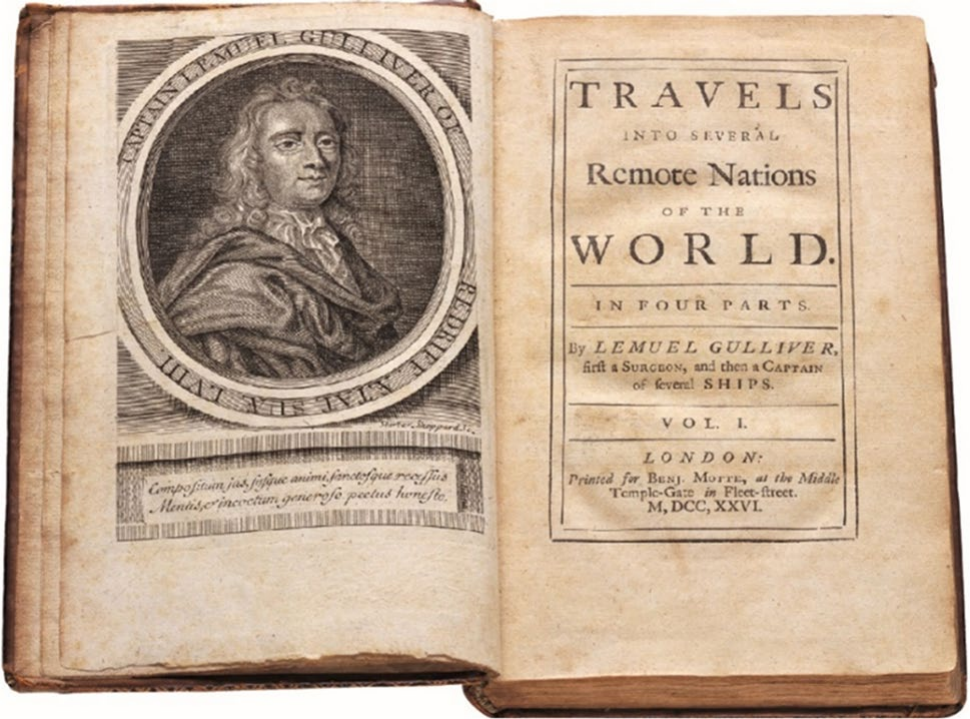
The original text of *Travels into several remote nations of the world* by Lemuel Gulliver. Picture on the *left* page is of Captain Lemuel Gulliver. Note that the author is Gulliver himself. Provided by courtesy of Meisei University Library, Tokyo, Japan

*Gulliver’s Travels* by Jonathan Swift, published in 1726, (Fig. 1) has gained wide recognition and popularity throughout the ages [1]. We are all excited by the adventures with the tiny (Lilliputian) and the gigantic (Brobdingnagian) fictitious human creatures that Gulliver encountered during his travels.

Recently, as a member of a book club, I had the chance to read the original text. I noticed by intuition an error regarding Gulliver’s estimated energy requirement in comparison to that of Lilliputians, tiny human creatures.

In the present study, the story of Gulliver was analyzed as an application material of scaling in comparative physiology. By the use of well-established equations, I have corrected the food requirements of Gulliver in the original text and further estimated heartbeats, respiration rates, life spans and blood pressures of the tiny (Lilliputian) and gigantic (Brobdingnagian) human creatures, whom Gulliver encountered during his travels to the fictitious islands they inhabited.

## Equations

Bibliographical analysis of *Gulliver’s travels* was made with the Planet ebook version available on the internet [1].

Physiological parameters of Gulliver and the fictitious human creatures are estimated using the following three equations:

1 $$W \propto H^{2} ,$$

2 $$E \propto W^{3/4} ,$$

3 $$T \propto W^{1/4} .$$

where *W*, *H*, *E*, and *T* denote body weight, height, energy consumption and physiological times, respectively.

## Body size

The height (*H*) of Gulliver was less than 6 feet, as described in *Gulliver’s Travels*:

*“…a strange creature [Gulliver] to be seen…, not so big as a splacnuck (an animal in that country very finely shaped, about six feet long,) and in every part of the body resembling a human creature…”* (Chapter II)

Galofré-Vilà et al. surveyed heights across the last 2000 years in England using femurs of skeletal remains [2]. According to this report, in the eighteenth century when Gulliver undertook the adventure, the height of males was 1.71 m on average.

Swift adopted the base of 12 in imagining these fictitious peoples, as seen below:

*“[His Majesty’s mathematicians] Having taken the height of my body by the help of quadrant and finding it to exceed theirs in the proportion of twelve to one…”* (Chapter III)

For Lilliputians, their size was 1/12 that of Gulliver, as stated in the text cited above. For the Brobdingnagians, their size can be deduced to be 12 times that of Gulliver, based on the following indirect statement:

*“…a hailstone is near eighteen hundred times as large as one in Europe.”* (Chapter V)

Supposing Gulliver is 1.71 m in height, the average for English males in the eighteenth century, the *H* of the Lilliputians and the Brobdingnagians can be estimated as 0.143 and 20.52 m, respectively (Table 1).

**Table 1:** Physiological parameters of Lilliputians, Gulliver and Brobdingnagians

	Lilliputian	Gulliver	Brobdingnagian
Body size			
*H* (height, m)	**0.143**	**1.71**	**20.52**
BMI (*W*/*H*^2^)	*23*	*23*	*23*
*W* (weight, kg)	0.47	67.3	9685
Energy			
Energy (*W*^3/4^) (relative ratios to Gulliver)	1/42	1	42
Calories required for daily life (kcal)	57	*2400*	100,800
Rate			
*W*^−1/4^(relative ratios to Gulliver)	3.5	1	1/3.5
Heartbeat (per min)	210	*60*	17
Breath (per min)	68	*18*	5
Time			
*W*^1/4^ (relative ratios to Gulliver)	1/3.5	1	3.5
Life span (years)	17	*60*	210
Blood pr.			
Blood pressure (mmHg)	*120*	*120*	*250–880*

Values shown in bold are indicated directly or indirectly in *Gulliver’s Travels*. Hypothetical values are shown in italic. The rest of the values are calculated using the equations shown above

Swift calculated weight (*W*) on the assumption that it is proportional to the cube of *H*. To the best of our knowledge, however, this is unlikely. As shown in Eq. 1, *W* is proportional to the square of *H*, not the cube of *H*. This principle was established in 1869 by Quetelet, a Belgian statistician, after a physical survey of tens of thousands of soldiers [3]. Based on this, the body mass index (BMI), which is defined by *W*/*H*^2^, was proposed and is now widely used as an index of obesity. It is also known, however, that Quetelet’s law is not applicable to outliers of *H*, including children, unless modifying the exponent of *H*.

In the present study, Quetelet’s law is applied to estimate *W* of tiny and gigantic creatures with extremely short and tall *H*, respectively, on the assumption that their body structures are geometrically similar to Gulliver as stated in the original text shown below, although nobody had looked at their real stature.

*“…a strange creature [Gulliver] … in every part of the body resembling a human creature [Lilliputians]…”* (Chapter II)

*“…His Majesty’s mathematicians concluded from the similarity of their bodies, that mine must contain at least 1724 of theirs*…” (Chapter III)

Assuming that each group has a BMI of 23, *W* for the Lilliputians, Gulliver and the Brobdingnagians will be 0.47, 67.3 and 9685 kg, respectively (Table 1). The sizes of the Lilliputians and the Brobdingnagians are close to those of rats and large dinosaurs, respectively.

## Energy consumption

Swift calculated energy consumption (*E*) simply based on *W* (or the cube of *H*), as described below:

*“[…the height of my body…exceed theirs in the proportion of twelve to one,] His Majesty’s mathematicians concluded from the similarity of their bodies, that mine must contain at least 1724 of theirs, and consequently would require as much food as was necessary to support that number of Lilliputians.”* (Chapter III)

(Note that, based on a figure of 12^3^, the value 1724 should be corrected to 1728).

**Fig. 2:**
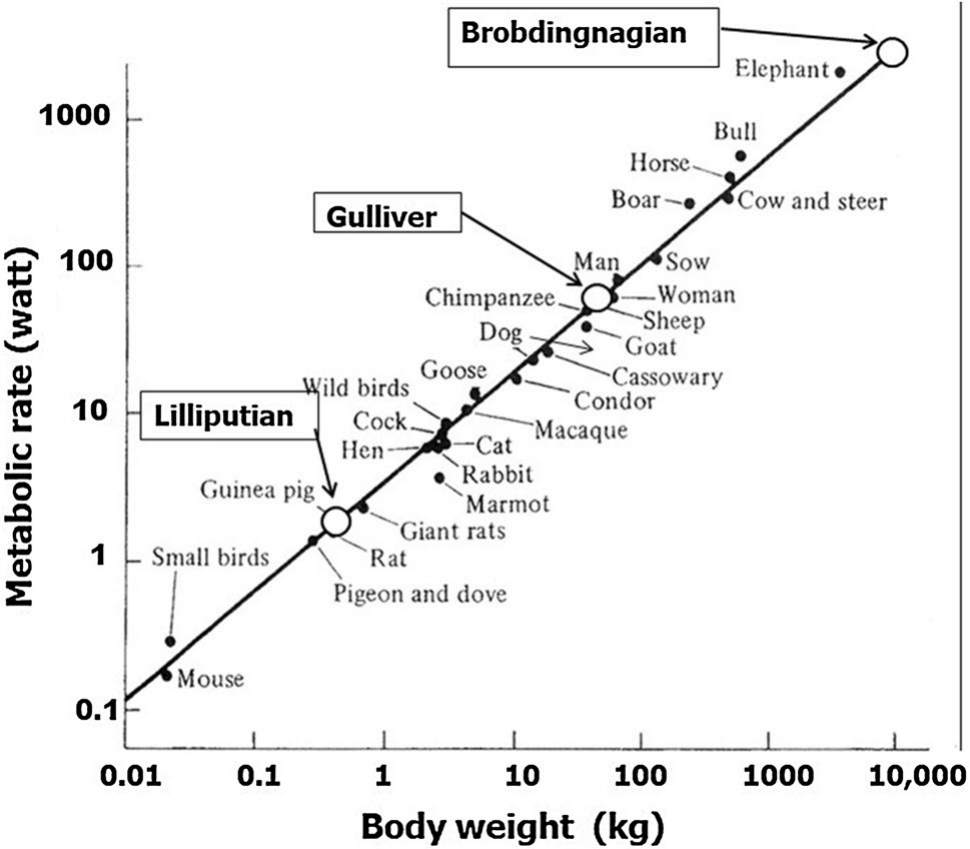
Energy (metabolic rate, watt) of various animals as a function of body mass (kg). Those of Lilliputians, Gulliver and Brobdingnagians are plotted on a regression line [5]

*E* is not simply proportional to *W*. A power law can be applied to *E* versus *W* with an exponent 3/4 (Eq. 2). When log *E* is plotted against log *W*, a linear regression line with a slope of 0.75 is apparent for a wide range of animals from small birds to the elephant (Fig. 2). This power law relationship, also called Kleiber’s law after his discovery of it in 1932 [4], can be utilized in estimating the food requirements of the peoples encountered by Gulliver.

By applying the above figures to the power law Eq. 2 and giving Gulliver the value of one, the relative ratios for energy required by the Lilliputians and the Brobdingnagians are 1/42 and 42, respectively. Thus, the food for 42 Lilliputians is sufficient to support Gulliver, rather than the 1724 suggested by Swift. It seems, therefore, that Swift greatly overestimated Gulliver’s food requirements.

These values also indicate that their daily calorific needs are approximately 57, 2400 and 100,800 kcal for the Lilliputians, Gulliver and the Brobdingnagians, respectively (Table 1). It is most likely that Gulliver gained much weight during his stay in Lilliput, lost it in Brobdingnag and eventually returned to the England with a normal body weight as a result of experiencing a balance between weight gain and loss.

## Heartbeats, breathing rate and life span

I further estimated times (*T*) related to vital physiological functions such as heartbeat and breath. These are also known to follow a power law with exponent 1/4 against *W* among species with a variety of values of *W* [5, 6]. For heartbeat and breathing rate, *T* indicates the time interval, so that their rates can be calculated reciprocally, i.e., with exponent of − 1/4. Ratios relative to Gulliver for heartbeat and breath are 3.5 and 1/3.5 for the Lilliputians and the Brobdingnagians, respectively. Assuming that Gulliver was healthy with a heartbeat of 60 beats/min and a breathing rate of 18/min, the corresponding rates for the Lilliputians would be 210 heartbeats and 68 breaths while those for the Brobdingnagians would be 17 and 5 (Table 1).

Life spans of most mammalian species also follow a power law with exponent 1/4 [6, 9], though human beings survive longer than other mammals with similar *W* values, probably due to having a bigger brain [5, 6]. Assuming that the brain size of the Lilliputians and the Brobdingnagians are proportional to that of Gulliver’s, their life spans would be 17 and 210 years, respectively, with Gulliver’s being estimated at 60 years (Table 1).

## Blood pressure

Quarter-power laws cannot be applied to blood pressure, which is known to be independent of body size, and which for most mammals is a constant 120 mmHg. This can be explained on the grounds of a consistent osmotic pressure among species resulting from a consistent plasma-protein concentration. One exception is the giraffe, for which the blood pressure is double that of a human being, i.e., 250 mmHg or more. This is necessary to supply sufficient blood to the brain via a 2-m-long neck [7]. Cardiologists speculate that barosaurus, a dinosaur with an extraordinarily long neck, must have maintained blood pressure of 880 mmHg in order to supply blood to the brain over a distance of 12 m from the heart [8]. It is speculated here that for the Brobdingnagian, with an estimated distance of some 5 meters from heart to brain (corresponding to 1/4 of *H*), the blood pressure would be more than that of a giraffe but less than that of a barosaurus, i.e., between 250 and 880 mmHg (Table 1).

## Previous studies

It is indeed surprising for me that nobody noticed this simple error for three centuries since publication in 1726. To the best of my knowledge, the only two exceptions are Max Kleiber, University of California at Davis, who published in 1967 a book chapter on this issue, but used misestimated values of Lilliputian height and did not take BMI into account [9 also cited in 10]. More recently, in 2014, A. J. Hulbert, University of Wollongong, Australia, published an article on Kleiber’s law, in which *Gulliver’s Travels* were briefly mentioned [11].

As for *H*, Kleiber used that of a Lilliputian page:

*“He [The Emperor of Lilliput] appeared to be of a middle age, and taller than any of the other three who attended him, whereof one was a page that held up his train, and seemed to be somewhat longer than my middle finger…”* (Chapter I)

Accordingly, Kleiber estimated 0.07 m as the *H* of a Lilliputian, whereas I used 0.143 m in the present study. As for *W*, Kleiber calculated by the cube of *H* as Swift did, rather than using Quetelet’s law (the square of *H*). Using these misread *H* and *W* values, Kleiber claimed that Gulliver was fed the foods equivalent to 675 Lillputians.

Hulbert [11] pointed out Kleiber’s mistake in *H* for Lilliputians by citing the same sentence as shown above from Swift’s original text. However, Hulbert mostly claimed limitation of the Kleiber’s law or 3/4 rule but without referring further to the food requirement of Gulliver in terms of that of the Lilliputians.

After my presentation, colleagues at the book club appreciated that they now had a better image of these fictitious peoples, but added that it was a unique but not necessarily proper viewpoint from which to consider this book. I agreed.
